# Skeletal stability after maxillary step osteotomy compared with original Le Fort I osteotomy during one-year of follow-up

**DOI:** 10.1038/s41598-019-46233-5

**Published:** 2019-07-05

**Authors:** Kazuto Kurohara, Nobuyoshi Tomomatsu, Koichi Nakakuki, Naoya Arai, Tetsuya Yoda

**Affiliations:** 1 Department of Maxillofacial Surgery, Division of Maxillofacial and Neck Reconstruction, Graduate School of Tokyo Medical and Dental University, Tokyo, Japan; 2 Department of Oral and Maxillofacial Surgery, Department of Clinical Sciences, Medical Life Science, Mie University Graduate School of Medicine, Mie, Japan

**Keywords:** Malocclusion, Fracture repair

## Abstract

The purpose of the current study was to compare the 1-year stability of skeletal after original Le Fort I osteotomy and maxillary step osteotomy. Fifty-two patients with prognathism underwent sagittal split ramus osteotomy with either original Le Fort I osteotomy or maxillary step osteotomy (26 patients each). Twelve cephalometric parameters were measured to evaluate postsurgical stability (lesser change was considered as enhanced stability) at 1 month (T1), 6 months (T2), and 1 year (T3) postoperatively. Only 3 parameters—vertical and horizontal distance of menton and vertical distance of point B—showed minimal but significant differences between the two groups. Lesser degrees of changes were observed after maxillary step osteotomy than after original Le Fort I osteotomy, and the differences were significant during the period between T1 and T2, but not from T1 to T3. Differences between the two groups were less in asymmetry cases required correction of the occlusal plane. In conclusion, differences between original Le Fort I osteotomy and maxillary step osteotomy were observed at the frontal points of the mandible; however, they were not clinically significant. It may be suggested that there is no significant difference in skeletal stability at 1 year after the two procedures.

## Introduction

Le Fort I osteotomy is the most widely used operative procedure to correct midfacial deformities. Reliable long-term results have been achieved, since the first description of the procedure by Wassmund in 1935^1^. Right-angled osteotomy lines have been proposed in order to overcome the disadvantages of linear osteotomy lines^2–6^. Maxillary step osteotomy technique, reported by Bennett and Wolford in 1985, is one of the modifications of the Le Fort I osteotomy^7^. The researchers described that the antero-posteriorly inclined linear osteotomy line of the conventional Le Fort I procedure can affect the stability of the repositioned maxillary segment depending upon the patterns of movement. During forward movement of the maxilla along the inclined osteotomy plane, the bony segment is moved in both forward and upward direction. During maxillary advancement independent of the osteotomy plane, a decrease in bony contact occurs at the final position and the maxillary segment is thought to be less stable^2^.

The method of bone fixation changed from wire to rigid fixation because some studies, which have used plates and screws for bone fixation, indicated lower relapse rates^8–10^. In addition, use of locking miniplate system for fixation of the maxilla became possible. However, under the systems of modern orthognathic surgery, the difference in postoperative stability of Le Fort I osteotomy between linear and step osteotomy techniques remains to be elucidated. The aim of the current study was to compare the skeletal stability after 1-year follow-up both original Le Fort I osteotomy and maxillary step osteotomy techniques after bimaxillary surgeries performed to correct mandibular prognathism and facial asymmetry.

## Results

### Variations in cephalometric parameters at each period - comparison of the stability between Group A and Group B

Group A (n = 26) comprised of 11 men and 15 women, and the average age of the patients was 24.7 years. Group B (n = 26) comprised of 11 men and 15 women, and the average age of the patients was 24.9 years. Variations in the cephalometric parameters (Table 1) at each period in Group A and Group B are shown in Table 2. Based on the examined cephalometric parameters (T0 to T1), no significant difference was observed between the two groups in jaw movement after the surgery. Postoperatively, no significant difference was observed in the variations of SNA, SNB, hA, hB, ramus inclination, vA, Gonial angle, MOC, and hMp, throughout the study period. Significant differences were observed in the variations of hM, vB, and vM from T1 to T2. Postoperatively, in Group A, five of the 26 patients (19.2%) had more than 2 mm change at point A in the sagittal direction (hA), as opposed to three of 26 patients (11.5%) in Group B (data not shown).

**Table 1:** Cephalometric parameters examined in this study.

Measurements	Description
Lateral	
*Sagittal relationships*	
SNA	Sella-Nasion to point A angle
SNB	Sella-Nasion to point B angle
hA	The perpendicular distance (mm) from point A to y-axis
hB	The perpendicular distance (mm) from point B to y-axis
hM	The perpendicular distance (mm) from Menton to y-axis
ramus inclination	Angle between the posterior line of the ramus and the FH line
*Vertical relationships*	
vA	The perpendicular distance (mm) from point A to x-axis
vB	The perpendicular distance (mm) from point B to x-axis
vM	The perpendicular distance (mm) from Menton to x-axis
Gonial angle	Angle between the posterior line of the ramus and the mandibular plane
Posterior-anterior	
*horizontal relationships*	
MOC	Angle between y axisis and line passing through Cg to the axisis origin point
hMp	The perpendicular distance (mm) from Menton to y-axis

**Table 2:** Variation of the cephalometric parameters in each period in Group A and Group B.

	T0-T1					T1-T2					T2-T3					T1-T3				
	Group A		Group B		*P*-value	Group A		Group B		*P*-value	Group A		Group A		*P*-value	Group A		Group B		*P*-value
	MD	SD	MD	SD		MD	SD	MD	SD		MD	SD	MD	SD		MD	SD	MD	SD	
SNA	1.45	2.12	2.13	1.29	0.351	−0.43	0.77	−0.57	0.81	0.680	−0.34	0.81	−0.13	0.66	0.361	−0.77	0.92	−0.71	0.85	0.647
SNB	−4.09	2.56	−3.16	2.33	0.315	0.02	0.97	−0.29	0.68	0.200	−0.02	0.75	0.14	0.68	0.421	0.00	0.90	−0.15	0.90	0.416
hA	1.97	3.03	2.77	1.69	0.347	−0.49	0.99	0.60	1.02	0.706	−0.45	1.10	−0.20	1.11	0.371	−0.94	1.23	−0.79	1.01	0.647
hB	−8.67	5.77	−6.75	4.95	0.375	0.35	2.22	−0.31	1.53	0.227	0.04	1.75	0.05	1.65	0.989	0.39	2.13	−0.26	1.59	0.224
hM	−10.98	6.29	−8.74	5.61	0.356	0.31	2.12	−0.94	2.15	0.026*	0.32	1.42	0.90	1.59	0.493	0.62	2.45	−0.04	2.46	0.432
ramus inclination	4.49	5.86	2.32	6.20	0.219	−1.39	3.45	1.71	3.54	0.528	−0.90	2.37	−0.48	2.69	0.631	−2.29	3.29	−2.19	3.02	0.903
vA	0.08	3.14	0.64	2.53	0.443	−0.18	1.33	0.04	0.82	0.504	−0.58	1.02	−0.25	1.26	0.324	−0.76	1.49	−0.20	1.24	0.235
vB	−2.43	4.02	−1.68	3.01	0.448	−1.43	1.15	−1.01	1.33	0.025*	−0.19	1.03	−0.18	0.95	0.768	−1.62	1.71	−1.18	1.44	0.061
vM	−2.56	3.49	−1.73	2.70	0.361	−1.02	1.14	−0.41	1.09	0.041*	0.06	0.80	−0.35	0.82	0.083	−0.96	1.34	−0.76	1.41	0.727
Gonial angle	−1.64	5.78	0.01	7.18	0.375	3.38	3.13	4.17	2.79	0.151	1.34	1.86	0.56	2.32	0.207	4.73	3.57	4.73	3.22	0.996
MOC	−1.92	2.06	−0.98	1.14	0.337	0.03	0.78	−0.10	0.73	0.351	−0.17	0.53	−0.04	0.52	0.552	−0.14	0.65	−0.14	0.67	0.831
hMp	−4.96	5.57	−2.55	2.94	0.220	−0.12	2.09	−0.16	1.86	0.768	−0.24	1.13	−0.18	1.34	0.931	−0.36	1.82	−0.34	1.71	0.867

T0, before surgery; T1, 1 month after surgery; T2, 6 months after surgery; T3, 1 year after surgery.

MD, mean difference; SD, standard deviation; *statistically significant (p < 0.05).

Horizontal changes, positive value indicates anterior movements and negative value indicates posterior mevement.

Vertical changes, positive value indicates inferior movements and negative value indicates superior movements.

MOC, hMp: positive value indicates diviation direction movements.

### Variations in cephalometric parameters at each period - comparison of the stability between Group AC and Group BC (cases without occlusal cant correction)

Group AC (n = 14) comprised of 7 men and 7 women, and the average age of the patients was 23.7 years. Group BC (n = 13) comprised of 4 men and 9 women, and the average age of the patients was 22.1 years. Variations in the cephalometric parameters at each period in Group AC and Group BC are shown in Table 3. Based on the examined cephalometric parameters (T0 to T1), no significant difference was observed between the two groups in jaw movement after the surgery. Postoperatively, no significant difference was observed in the variations of SNA, SNB, hA, hB, ramus inclination, vA, vM, Gonial angle, MOC, and hMp throughout the study period. Significant differences were observed in the variations of hM, and vB from T1 to T2. Significant differences were also observed in the variations of vB from T1 to T3.

**Table 3:** Variation of the cephalometric parameters in each period in Group AC and Group BC (cases without occlusal cant correction).

	T0-T1					T1-T2					T2-T3					T1-T3				
	Group AC		Group BC		*P*-value	Group AC		Group BC		*P*-value	Group AC		Group BC		*P*-value	Group AC		Group BC		*P*-value
	MD	SD	MD	SD		MD	SD	MD	SD		MD	SD	MD	SD		MD	SD	MD	SD	
SNA	1.70	1.46	2.64	1.25	0.169	−0.31	0.93	−0.54	0.59	0.369	−0.42	0.93	0.00	0.57	0.224	−0.73	0.97	−0.54	0.78	0.659
SNB	−4.62	2.32	−3.56	1.63	0.198	0.18	0.90	−0.26	0.72	0.137	−0.01	0.72	0.33	0.32	0.125	0.17	0.94	0.07	0.79	0.356
hA	2.26	2.23	3.55	1.52	0.274	−0.37	1.22	−0.82	0.82	0.224	−0.47	1.16	0.08	0.84	0.119	−0.84	1.29	−0.74	1.03	0.858
hB	−10.07	5.41	−7.32	3.74	0.202	0.74	2.03	−0.49	1.73	0.059	0.11	1.52	0.41	0.91	0.480	0.85	1.96	−0.09	1.36	0.102
hM	−12.13	5.77	−9.71	4.65	0.259	0.99	1.73	−1.00	2.26	0.034*	0.61	1.42	0.92	1.31	0.526	1.60	2.31	−0.09	2.43	0.087
ramus inclination	4.95	5.10	3.98	6.85	0.550	−1.45	3.74	−2.41	4.42	0.422	−0.77	2.05	0.23	2.23	0.220	−2.22	3.56	−2.18	3.41	0.820
vA	0.43	3.36	0.60	3.01	0.971	0.00	1.40	0.24	0.81	0.558	−0.79	1.14	−0.19	1.01	0.141	−0.79	1.80	0.05	1.00	0.244
vB	−2.31	3.86	−2.33	3.59	0.981	−1.72	1.06	−0.77	1.31	0.011*	−0.49	0.56	−0.42	0.91	0.895	−2.21	0.97	−1.20	1.61	0.019*
vM	−2.42	3.30	−2.49	3.11	0.858	−1.07	0.85	−0.12	1.29	0.056	−0.11	0.61	−0.27	0.87	0.496	−1.18	1.02	−0.39	1.70	0.181
Gonial angle	−3.10	5.42	−1.57	8.32	0.583	3.66	3.49	4.49	3.10	0.102	1.57	1.91	0.35	1.17	0.083	5.23	3.49	4.84	3.43	0.981
MOC	−0.90	1.73	−0.37	0.98	1.000	−0.13	0.61	−0.02	0.90	0.659	−0.10	0.25	−0.14	0.65	0.802	−0.22	0.61	−0.15	0.79	0.729
hMp	−2.23	4.68	−1.04	2.53	0.711	−0.45	1.90	0.08	2.26	0.933	−0.24	0.74	−0.41	1.61	0.685	−0.69	1.79	−0.34	2.02	0.574

T0, before surgery; T1, 1 month after surgery; T2, 6 months after surgery; T3, 1 year after surgery.

MD, mean difference; SD, standard deviation; *statistically significant (p < 0.05).

Horizontal changes, positive value indicates anterior movements and negative value indicates posterior mevement.

vertical changes, positive value indicates inferior movements and negative value indicates superior movements.

MOC, hMp; positive value indicates diviation direction movements.

### Variations in cephalometric parameters at each period - comparison of the stability between Group AD and Group BD (cases with occlusal cant correction)

Group AD (n = 12) comprised of 4 men and 8 women, and the average age of the patients was 25.8 years. Group BD (n = 13) comprised of 7 men and 6 women, and the average age of the patients was 27.8 years. Variations in the cephalometric parameters at each period in Group AD and Group BD are shown in Table 4. Based on the cephalometric parameters, significant differences were observed between the two groups in jaw movement after the surgery (T0 to T1). Significant differences were observed in the variations of MOC and hMp. No significant difference was observed in the variations of SNA, SNB, hA, hB, hM, ramus inclination, vA, vB, vM, and Gonial angle. Postoperatively, no significant difference was observed in the variations of SNA, SNB, hA, hB, hM, ramus inclination, vA, vB, vM, Gonial angle, MOC, and hMp throughout the study period.

**Table 4:** Variation of the cephalometric parameters in each period in Group AD and Group BD (cases with occlusal cant correction).

	T1-T0					T2-T1					T3-T2					T3-T1				
	Group AD		Group BD		*P*-value	Group AD		Group BD		*P*-value	Group AD		Group BD		*P*-value	Group AD		Group BD		*P*-value
	MD	SD	MD	SD		MD	SD	MD	SD		MD	SD	MD	SD		MD	SD	MD	SD	
SNA	1.16	2.75	1.62	1.15	0.989	−0.57	0.53	−0.61	1.01	0.649	−0.26	0.68	−0.27	0.74	0.841	−0.82	0.91	−0.87	0.91	0.925
SNB	−3.48	2.78	−2.75	2.89	0.728	−0.17	1.06	−0.32	0.67	0.926	−0.03	0.81	−0.05	0.89	0.779	−0.20	0.86	−0.37	0.98	0.698
hA	1.63	3.83	2.00	1.53	0.718	−0.63	0.66	−0.38	1.17	0.313	−0.43	1.07	−0.47	1.31	0.841	−1.06	1.22	−0.85	1.03	0.621
hB	−7.04	5.97	−6.17	6.02	0.967	−0.10	2.43	−0.13	1.35	0.678	−0.04	2.06	−0.31	2.14	0.650	−0.14	2.28	−0.44	1.82	0.780
hM	−9.63	6.85	−7.78	6.48	0.810	−0.49	2.33	−0.88	2.13	0.574	−0.03	1.41	0.88	1.88	0.461	−0.52	2.16	0.00	2.60	0.503
ramus inclination	3.96	6.85	0.65	5.21	0.270	−1.33	3.23	−1.01	2.34	0.852	−1.04	2.79	1.18	3.00	0.538	−2.37	3.09	−2.19	2.72	1.000
vA	−0.33	2.96	0.68	2.06	0.106	−0.39	1.26	−0.15	0.81	0.601	−0.33	0.83	−0.31	1.51	0.968	−0.73	1.09	−0.46	1.43	0.948
vB	−2.58	4.36	−1.02	2.23	0.288	−1.09	1.21	−1.24	1.35	0.883	0.16	1.35	0.07	0.95	0.925	−0.93	2.14	−1.17	1.32	0.739
vM	−2.72	3.85	−0.98	2.07	0.140	−0.97	1.45	−0.70	0.79	0.288	0.27	0.96	−0.43	0.79	0.066	−0.70	1.65	−1.13	0.97	0.494
Gonial angle	0.06	5.95	1.59	5.70	0.565	3.06	2.76	3.85	2.54	0.461	1.08	1.86	0.77	3.12	0.990	4.14	3.74	4.62	3.13	0.904
MOC	−3.11	1.81	−1.59	0.97	0.046*	0.21	0.94	−0.18	0.53	0.397	−0.25	0.74	0.05	0.36	0.354	−0.04	0.72	−0.14	0.56	0.820
hMp	−8.15	4.92	−4.06	2.59	0.040*	0.26	2.33	−0.39	1.41	0.905	−0.23	1.50	0.05	1.02	0.936	0.03	1.86	−0.34	1.41	0.495

T0, before surgery; T1, 1 month after surgery; T2, 6 months after surgery; T3, 1 year after surgery.

MD, mean difference; SD, standard deviation; *statistically significant (p < 0.05).

horizontal changes, positive value indicates anterior movements and negative value indicates posterior movement.

vertical changes, positive value indicates inferior movements and negative value indicates superior movements.

MOC, hMp; positive value indicates diviation direction movements.

## Discussion

The maxillary step osteotomy technique is a modification of Le Fort I osteotomy. A major advantage of the step osteotomy is that it can provide pure anteroposterior maxillary movements^7^. The main focus of the current study was to analyze the probable advantages of the step osteotomy technique in postoperative stability.

Prior to evaluation of the difference between LO and SO, skeletal stability of the Le Fort I osteotomy done in the current study was compared with those in previous studies. The present study showed that mean postoperative changes at point A were less than 1 mm, both horizontally and vertically (differences in hA and vA between T1 and T3). Horizontal changes (differences in hA between T1 and T3) were −0.94 mm (mean, SD = 1.23) in the LO group, and −0.79 mm (mean, SD = 1.01) in the SO group. Previous studies have reported that postoperative changes greater than 2 mm are clinically significant and are considered as skeletal relapse^11–14^. Dowling *et al*.^15^ reported that skeletal relapse (≧2 mm) occurred in 14% of the patients who underwent Le fort I advancement. Chen *et al*.^16^ reported that maxillary relapse occurred in 16.7% of the included patients. In the present study, relapse (changes exceeding 2 mm) was observed in 19.2% (5 of the 26) and 11.5% (3 of the 26) patients in LO and SO groups, respectively. Meanwhile, vertical changes (differences in vA between T1 and T3) were −0.76 mm (mean, SD = 1.49) in the LO group, and 0.20 mm (mean, SD = 1.24) in the SO group. Politi *et al*.^17^ reported that the mean postoperative vertical change was −0.24 mm at point A in patients who underwent combined maxillary advancement and mandibular setback with rigid internal fixation. The maxillary postoperative changes seen in the patients in the present study were comparable to those in the previous studies^15–17^, indicating that the stability of repositioned maxilla was the same, regardless of the method of osteotomy.

Mandibular postoperative stability in the present study was also comparable to a previous report by Park *et al*.^18^. The setback distances of the LO and SO groups in the current study were approximately 8.67 mm and 6.75 mm, respectively (differences in hB between T0 and T1). Postoperative horizontal changes (differences in hB between T1 and T3) were as minimal as 0.395 mm in LO and −0.264 mm in SO, that represented 4.56% and 3.9% of the above setback distance, respectively. Park *et al*.^18^ studied 20 cases combining Le Fort I maxillary with bSSRO mandibular setback using rigid fixation and showed that the average horizontal relapse was 0.85 mm of 7.72 mm mean setback distance, representing a relapse rate of 11.1%. Mean vertical movements of the mandible measured at point B were 2.43 mm in the LO group and 1.68 mm in the SO group. Postoperative changes (differences in vB between T1 and T3) were 1.62 mm and 1.18 mm that represented 66.6% and 70.2% of the above movements, respectively. In a study by Park *et al*.^18^, wherein conventional bimaxillary surgery was performed, the average vertical relapse was 2.744 mm of 4.096 mm mean superior movement, representing a relapse rate of 67.0%. Based on the above observations, it was considered that the stability of the postoperative mandibular position in the current study was similar to that of the previous study.

To investigate the probable difference in skeletal stability after conventional linear osteotomy and modified step osteotomy, the above-mentioned horizontal and vertical changes were compared between the LO and SO groups. Statistically significant differences were observed between the two groups in the measurements of hM, vB, and vM, in the early postoperative period (within 3 months postoperatively). The differences in hM, vB, and vM were 1.25 mm, 0.42 mm, and 0.61 mm, respectively. However, the differences were not consistently observed throughout the duration of follow-up (up to 1 year postoperatively). Thus, the difference between LO and SO was minor and temporary to be clinically significant^11–14^. There was limited clarity regarding the occurrence of statistical differences between the two groups in the early postoperative period alone, as well as the difference being observed only in the mandible. Pertaining to the latter observation, it may be speculated that slight maxillary clockwise or anticlockwise rotation might be amplified in the mandible. Lee *et al*.^19^ studied skeletal stability after modified quadrangular Le Fort I osteotomy (MQLI), and no statistically significant of skeletal stability was reported between LO and MQLI.

The current study also examined the probable influence of occlusal cant correction on the stability in both groups. Statistically significant difference between LO and SO was observed only in the group without occlusal cant correction (Group AC and Group BC). In the group without occlusal cant correction, the difference (1.01 mm) in vB was statistically significant during the entire duration of follow-up. Without occlusal cant correction, the variation in the measurement of vB is small because the direction of the relapse is simple, and the difference between LO and SO may be reflected to cause a significant difference in the amount of post-operative change in vB. In orthognathic surgery, osteosynthesis devices^17,19^ have been developed since Bennett and Wolford reported the maxillary step osteotomy technique^7^. Therefore, it is possible that the disadvantages of the conventional Le Fort I osteotomy may have been improved by use of structurally strong miniplates and locking type miniplates^20^.

The current study is associated with some limitations. First, only two-dimensional skeletal changes were assessed with the use of cephalometric radiographs. Ideally, computed tomography (CT) should be utilized to measure the three-dimensional skeletal changes. Second, the sample size in the present study was small and the design was retrospective in nature. Third, homogeneity of patients and the surgical technique are fundamental for critical analysis of the multi-factorial nature of postoperative changes^21^. In the present study, the same surgeon performed the surgeries and all patients underwent Le Fort I osteotomy and sagittal split ramus osteotomy stabilized with titanium plates and screws, although the plates and screws were provided by two companies. However, multiple orthodontists performed the orthodontic treatment. Forth, the observation was a 1-year follow-up period in the present study. Profitt *et al*. described the period of follow-up for Class III patients as: short-term (1 year) and long-term (1 to 5 years)^22^. Previous studies have indicated that relapse was observed after 2 years of follow-up^23,24^. Long-term (1–5 years) stability after LO vs SO in bimaxillary surgery needs to be evaluated in the future.

In conclusion, statistically significant differences were observed between the original Le fort I osteotomy and step osteotomy techniques at the point B and menton; however, the changes were minimal to be clinically significant. Although there was limitation to evaluate the stability after LO vs SO for Class III over the long-term follow-up, the results suggest that both procedures are not associated with significant difference in the skeletal stability after 1-year follow-up.

## Material and Methods

### Patients

**Figure 1:**
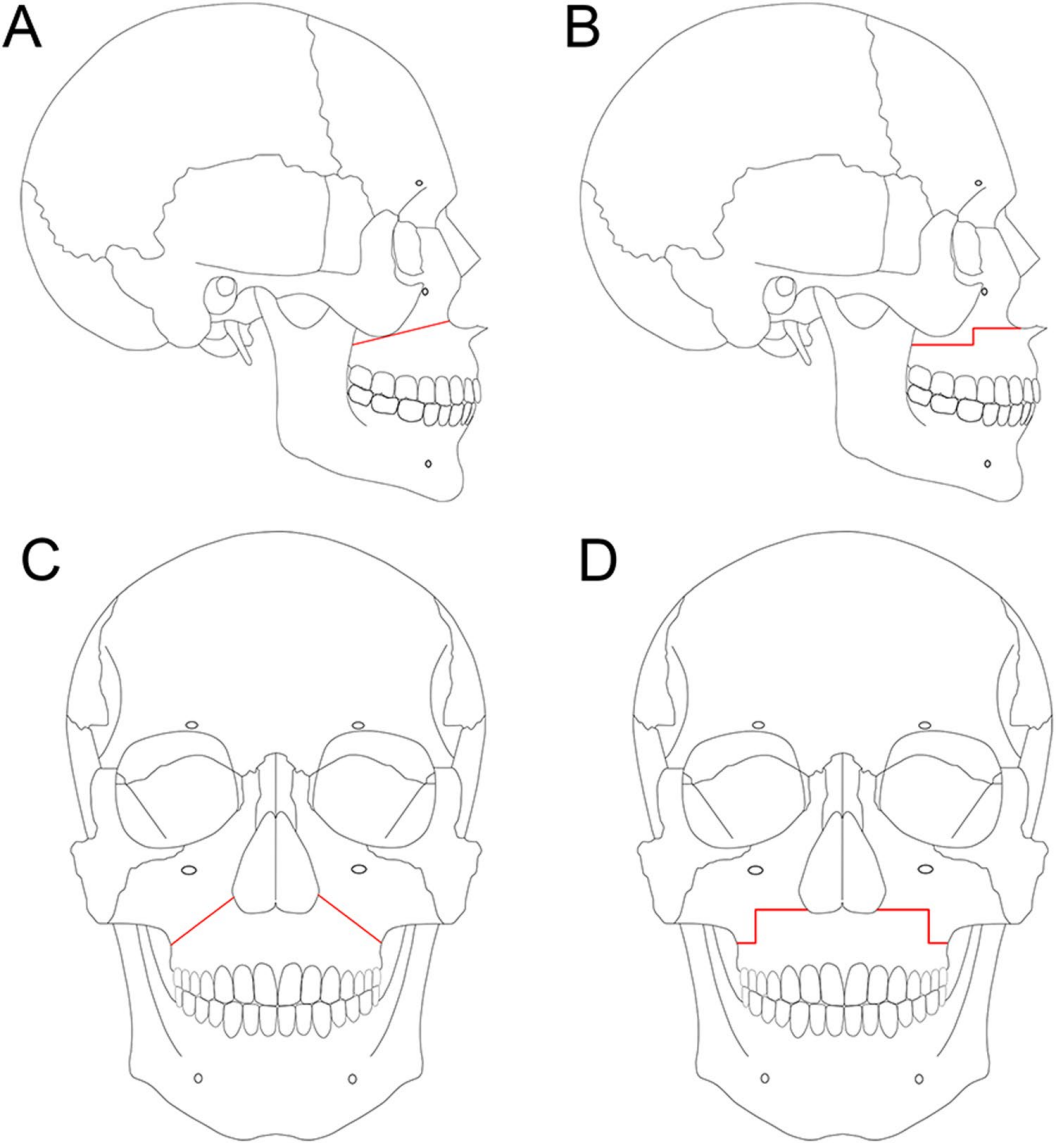
Variations in Le Fort I osteotomy. (**A**) Lateral view of original Le Fort I osteotomy. (**B**) Lateral view of maxillary step osteotomy. (**C**) Frontal view of original Le Fort I osteotomy. (**D**) Frontal view of maxillary step osteotomy.

This retrospective study comprised of 52 patients with mandibular prognathism. All patients underwent Le Fort I osteotomy in combination with bilateral sagittal split ramus osteotomy (bSSRO) using rigid internal fixation (RIF) at the Tokyo Medical and Dental University Dental Hospital, Tokyo, Japan, from January 2010 to December 2015. The patients did not have any genetic syndromes or other congenital deformities. In this study, the original Le Fort I osteotomy was performed for the patients from 2010 to 2014 (26 cases), and the step osteotomy was performed from 2013 to 2015 (26 cases). For uniformity of the study groups, the same number of patients was included in the two groups: 26 patients were treated by original Le Fort I osteotomy (LO) (Group A) and the other 26 patients by step osteotomy (SO) (Group B) (Fig. 1). As it was possible that correction of the cant of the occlusal plane (so called, “rolling”) might affect the postoperative skeletal stability, the patients were further assigned into four subgroups, LO without (Group AC, n = 14), and with (Group AD, n = 12) occlusal cant correction, and SO without (Group BC, n = 13), and with (Group BD, n = 13) occlusal cant correction. The patients were assigned into the subgroup ‘with occlusal cant correction’ if the value was greater than 2 degrees. Pubertal growth of all patients was complete. One senior maxillofacial surgeon performed the surgery of this study. Patients with craniofacial deformities, previous trauma to the facial skeleton, and class II malocclusion were excluded from the study. The Institutional Review Board (IRB) of the Tokyo Medical and Dental University (D2017-075) approved the current study. All methods were performed in accordance with the relevant guidelines and regulations. This study is in accordance with the ethical standards of the institutional and/or national research committee and with the 1964 Helsinki declaration and its later amendments or comparable ethical standards. Informed consent for participation was obtained in the form of opt-out on the web site.

### Surgery

The original Le Fort I osteotomy were performed as described in a study by Bell^25^. The maxillary step osteotomy was performed as described in the study by Bennett and Wolford^7^. The surgical procedure performed in groups A and B were same, barring the difference in the osteotomy lines and manufacturers of the miniplates used (Fig. 1). The new position of maxilla was verified using occlusal splints. In all cases, the maxilla was rigidly osteosynthesized with titanium miniplates and screws at the zygomatic buttress and the pyriform aperture. Rigid fixation with titanium miniplates and screws was used to stabilize the maxilla and mandible. Titanium miniplate system (KLS Martin, Tuttlingen, Germany) with non-locking screws or titanium miniplate MOJ system (DePuySynthes Johnson & Johnson K.K., Pennsylvania, the United States of America) with locking screws was used for fixation in the current study. Postoperative intermaxillary fixation with wiring was not performed. All patients underwent orthodontic therapy before and after surgery.

### Cephalometric analysis

**Figure 2:**
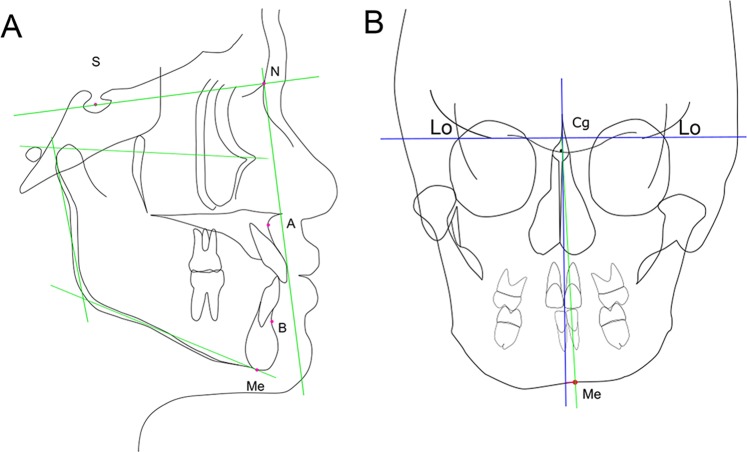
Cephalometric reference points and lines. (**A**) Lateral view. S, sella; N, nasion; A, point A; B, point B; Me, menton; x-axis, line passing through sella to nasion; y-axis, line passing through nasion and perpendicular to x-axis. (**B**) Frontal view. Lo, intersection of lateral margin of orbit and medial margin of temporal fossa; Cg, middle point of neck of crista galli; Me, menton; x-axis, line passing through right and left latero-orbitale point; y-axis, line passing through crista galli and perpendicular to x-axis.

Lateral and frontal cephalometric radiographs were used to analyze and measure the angle and distance (Fig. 2, Table 1). The radiographs were taken before surgery (T0) and at 1 month (T1), 3 months (T2), and 1 year (T3) after surgery. Cephalograms were analyzed using the measuring software ApolloViewLite version 4.16.8.2 (Simono Osamu, Japan). The following anatomic points were identified on the cephalogram: N, nasion; S, sella turcica; A, point A; B, point B; Gn, gnathion; Me, menton; and Cg, crista galli (Fig. 2). The S-N plane was considered the x-axis for the vertical measurements, whereas the y-axis for the horizontal measurements was drawn perpendicular to the x-axis passing point N, on the lateral cephalogram (Fig. 2A). Maxillary and mandibular skeletal landmarks were used to analyze stability. The maxillary skeletal landmark was the point A. The perpendicular distance from the y-axis to point A was measured to evaluate the horizontal movement. The mandibular skeletal landmarks were point B and menton. The perpendicular distance from the y-axis to point B and menton was measured to evaluate the horizontal movement. Ramus inclination is the angle between the posterior line of the ramus (articular point-ramus down point) and the FH line. On the frontal cephalogram, the line connecting the left and right latero-orbitale point was considered the x-axis, and the y-axis was drawn perpendicular to the x-axis passing through the crista galli point (Fig. 2A). Menton inclination (MOC) is the angle between the y-axis and the line passing through the menton and the intersection point of the x and y-axes. The MOC angles and hMp distances were given positive values to the mandibular deviated side on the frontal cephalogram. The reference points and variables used are presented in Table 1. The angular measurements were recorded in degrees and the linear measurements were recorded in millimeters. The researcher measured each parameter (each distance and angle) thrice and the average was represented for each measurement.

### Statistical analyses

Data analyses including calculation of descriptive statistics were carried out using the statistical software R version 3.5.1 (R Core Team. R Foundation for Statistical Computing, Vienna, Austria. URL https://www.R-project.org/). The Wilcoxon rank-sum test was used to compare the cephalometric measurements between the groups to evaluate the changes at the different time points. Differences were considered significant at *p*-value of < 0.05.

## Data Availability

The datasets generated during and/ or analyzed during the current study are available from the corresponding author on reasonable request.
